# Severe/Extreme Hypertriglyceridemia and LDL Apheretic Treatment: Review of the Literature, Original Findings

**DOI:** 10.1155/2014/109263

**Published:** 2014-12-16

**Authors:** Olga Diakoumakou, Georgios Hatzigeorgiou, Nikos Gontoras, Maria Boutsikou, Vana Kolovou, Sophie Mavrogeni, Vassiliki Giannakopoulou, Genovefa D. Kolovou

**Affiliations:** ^1^Cardiology Department, Onassis Cardiac Surgery Center, 356 Sygrou Avenue, 176 74 Athens, Greece; ^2^Molecular Immunology Laboratory, Onassis Cardiac Surgery Center, 356 Sygrou Avenue, 176 74 Athens, Greece

## Abstract

Hypertriglyceridemia (HTG) is a feature of numerous metabolic disorders including dyslipidemias, metabolic syndrome, and diabetes mellitus type 2 and can increase the risk of premature coronary artery disease. HTG may also be due to genetic factors (called primary HTG) and particularly the severe/extreme HTG (SEHTG), which is a usually rare genetic disorder. Even rarer are secondary cases of SEHTG caused by autoimmune disease. This review considers the causes of SEHTG, and their management including treatment with low density lipoprotein apheresis and analyzes the original findings.

## 1. Introduction

A positive correlation between high triglycerides (TGs) concentration and coronary heart disease (CHD) has been established in numerous studies [1–11]. Hypertriglyceridemia (HTG) is prevalent in 18.6% of men and 4.2% of women between 16 and 65 years of age. The Adult Treatment Panel (ATP) III guidelines, published 13 years ago [12], described normal TGs concentration <150 mg/dL (<1.6 mmol/L), borderline-high TGs as 150 to 199 mg/dL (1.6–2.2 mmol/L), high TGs as 200 to 499 mg/dL (2.2–5.6 mmol/L), and very high TGs as >500 mg/dL (>5.6 mmol/L).

However, severe/extreme hypertriglyceridemia (SEHTG) should be considered when values are greater than 1,000 mg/dL (11.2 mmol/L) because this places individuals at significant increased risk of pancreatitis. With TG values less than 1,000 mg/dL (5.6 mmol/L) one should be focused on the risk of premature CHD [13].

HTG is a feature of numerous metabolic disorders including dyslipidemias, metabolic syndrome, and diabetes mellitus type 2 (DMT2) and can increase the risk of premature CHD [14, 15]. These metabolic disorders may be caused by interactions between genetic and nongenetic factors since those subjects present usually similar clinical features (android type of obesity, ectopic fat deposition, thin arms and legs, increased waist circumference, upper body obesity, and in case of SEHTG eruptive xanthomas) [16, 17]. Visceral fat is considered to behave as ectopic fat deposition. It accumulates TGs in cases when body fat storage exceeds the capacity of fat stores. Furthermore, subjects with HTG usually present insulin resistance, hepatic steatosis, and DMT2. Thus, all the above can be called “hypertriglyceridemic phenotype.”

Additionally, several studies (including ours) showed that postprandial HTG is manifested in subjects with hypertriglyceridemic phenotype [18].

HTG may also be due to genetic factors (called primary HTG) and particularly the SEHTG, which is a usually rare genetic disorder. Even rarer are secondary cases of SEHTG caused by autoimmune disease.

The most severe complication of SEHTG is acute pancreatitis, which is associated with high mortality and morbidity reaching approximately 10% of affected patients [19]. The SEHTG is the 3rd common cause of acute pancreatitis after alcoholism and cholelithiasis. SEHTG is reported to be responsible for 10% of acute pancreatitis occurrence [20]. In severe form of acute pancreatitis the development of pancreatic necrosis, infection, and multisystem organ failure (respiratory, renal, hepatic, and cardiovascular) is observed.

Chylomicrons (CMs), which are presented in the SEHTG, are large enough to obstruct the pancreatic capillaries leading to pancreatic ischemia and release of pancreatic lipase. As a consequence of that the enhanced lipolysis leads through increased concentration of the free fatty acids in the circulation to vascular endothelial cell damage [21] and chaotic cellular membrane trafficking, which starts a cascade of deleterious effects (conversion of trypsinogen to trypsin, activation of zymogen cascade, activation of inflammatory cells, and release of cytokines that further mediate local inflammatory responses). All the above described modifications are leading to hemorrhage, edema, and eventually pancreatic necrosis. Furthermore, as the mediators are passing into the circulation, other complications can develop such as bacteremia, acute respiratory distress syndrome, renal failure, and gastrointestinal hemorrhage. The systemic inflammatory response syndrome can also develop, leading to the development of systemic shock and death.

On the other hand, the management of severe acute pancreatitis is problematic because of multicausality of disease and a few effective treatment protocols. The treatment of acute pancreatitis is beyond the scope of this review. Generally, sterile pancreatic necrosis can be managed conservatively; infection of pancreatic necrosis can be managed by removing nonvital tissue to control the sepsis (early surgical intervention may lead to worse prognosis) and the support of failure organ should be performed.

This review considers the causes of SEHTG and their management including treatment with low density lipoprotein (LDL) apheresis and analyzes the original findings according to LDL apheresis treatment.

## 2. TGs Metabolism

### 2.1. Fasting State

The circulating plasma TGs are formed mainly from two major sources: intestinally derived chylomicrons (CMs) and hepatically derived very low density lipoproteins (VLDL) [22]. In the intestinal cells, dietary TGs together with dietary cholesterol are packed and, after binding to apolipoprotein (apo) B-48 (mediated by microsomal triglyceride transport protein, MTP), the CMs are formed, which are then transported via lymphatic vessels-blood circulation to other cells of the body [23, 24]. In the hepatic cells the newly synthetized TGs are binding to apo B-100, also mediated by MTP, and formed VLDL particles which are released to blood circulation. In the vascular wall the lipoprotein lipase (LPL, found on the endothelial surfaces of capillaries) hydrolyses TGs from VLDL particles and remnants, intermediate density lipoproteins (IDLs), are formed which are further hydrolyzed to LDL particles. The TGs from CMs are also hydrolyzed by LPL to free fatty acids and glycerol, which are taken up by muscle cells and adipose tissue [25] for depots. The remaining CMs formed CM remnants and undergo further hydrolysis by LPL with apo C-II as a cofactor and enter the liver (in the space of Disse) where endocytosis takes place. The endocytosis of CM remnants could be via the LDL receptors (LDLR) and via LDLR related protein (LRP) and via other several hepatic receptors. Also, the VLDL receptor (VLDLR), member of the LDLR supergene family, has been recognized as a physiological receptor for CM remnants.

The formation of VLDL particles in the liver depends on the fatty acid supplies, hepatic TGs pools, and cholesterol substrate supplies to the liver [26]. For example, high availability of cholesterol substrate reduces hepatic cholesterol synthesis; the lower secretion of VLDL particles is followed by the upregulation of LDLRs. The removal of VLDL particles follows the same pathway as the CM remnants.

### 2.2. Nonfasting State

The rate of dietary TGs clearance is the result of several variables. Particularly, the size of capillaries bed, the amount of active LPL, and the competition between VLDL and CM particles are the main factors that influence this clearance.

Under the normal conditions, the CM and VLDL remnants are briskly removed from the circulation by a receptor-mediated process and are not converted into smaller, lower density particles. Xiang et al. have shown that the removal of postprandial TGs from CMs is 10 times greater than that from VLDL particles [27]. Besides that, the number of VLDL particles is much higher than that of CMs (about 20 : 1) and the increase in VLDL particle number in the postprandial state is greater than the increase in CMs. In the early postprandial period (the first 3 h after a meal) smaller sized CMs are secreted. Later in the postprandial period, de novo-formed larger CMs are secreted. The smaller sized CMs are considered to be atherogenic. In healthy subjects, VLDLs secreted by the liver are not considered to be atherogenic.

Generally, during postprandial lipemia, the cholesteryl ester transfer protein- (CETP-, glycoprotein secreted mainly from the liver) mediated transfer of cholesteryl esters and TGs between plasma lipoprotein particles is increased, allowing transformation of cholesteryl ester-enriched HDL into TG-rich HDL particles which become a substrate for hepatic lipase and are cleared more rapidly from the circulation.

## 3. HTG

In the environments of high TGs concentration the transfer rate of cholesteryl esters from HDL to VLDL is elevated, resulting in the secretion of large VLDL particles. These particles are typically catabolized to the small dense LDL particles and are removed by scavenger receptors (non-LDLR-mediated pathway). When the level of VLDLs is within the normal range, the CETP-mediated transfer of HDL cholesteryl esters is directed preferentially to LDL particles. When the concentration of VLDL particles is increased, the cholesteryl esters of HDL are preferentially transferred by CETP to larger VLDL particles.

## 4. SEHTG

The causes of SEHTG can be defined as primary or secondary. The primary causes of SEHTG are rare (<10% of cases) and often monogenic in etiology and frequently cause pancreatitis such as familial chylomicronemia, LPL deficiency, familial apolipoprotein (apo) C-II deficiency, and primary hyperlipemia type V. Secondary causes include DMT2, MetS, chronic renal failure, nephrotic syndrome, hypothyroidism, pregnancy, excess alcohol intake, and medications such as corticosteroids, estrogens, retinoids, diuretics, and antiretroviral therapy.

Since the excess alcohol intake is one of the more frequent secondary causes of acute pancreatitis the short analysis will be provided. There is considerable evidence that excess alcohol intake has been associated with an increase in TG concentration and risk of pancreatitis [28]. However, the association between alcohol and TG concentration is complex and does not fit to linear association but follows the J-shaped association [29]. Alcohol-induced HTG is due to increased VLDL secretion, impaired lipolysis, and increased free fatty acid fluxes from adipose tissue to the liver. Noteworthy to mention is that low to moderate alcohol consumption may be associated with decreased TG concentration; see review by Kolovou et al. [30]. However, patients should be recommended to reduce or stop alcohol consumption in case of HTG.

Some of more frequent primary SEHTG will be briefly analyzed below.

### 4.1. Familial Chylomicronemia

The diagnosis of familial chylomicronemia depends on the presence of elevated plasma TG (>2,000 mg/dL), which can reach 20,000–30,000 mg/dL in rare cases, along with characteristic clinical symptoms or signs, such as abdominal pain/acute pancreatitis and eruptive xanthomas, lipemia retinalis, and hepatosplenomegaly. In addition, reduced levels of HDL cholesterol are observed, while plasma presents milky tint. The disease is habitually present in patients suffering from further immune diseases, like idiopathic thrombocytopenic purpura and Graves' disease.

### 4.2. Familial LPL Deficiency

Familial LPL deficiency is an autosomal recessive inherited disease caused by mutations in the* LPL* gene and is clinically expressed as chylomicronemia syndrome. LPL deficiency may demonstrate in early childhood acute pancreatitis or abdominal or colicky pains, eruptive xanthomas (formed usually on the buttocks, elbows, back, and knees and regress with lowering TGs concentration), lipemia retinalis, and hepatosplenomegaly [31]. If it presented shortly after the birth it may lead to failure to thrive, irritabilities, and diarrhea. The plasma of these patients is always milky white or lipemic due to 10 to 100 times increased TG concentration, even under fasting conditions. The HDL concentration is evidently decreased. This clinical complication can be lethal. Complications occur when TGs concentration is >1,000 mg/dL [32].

### 4.3. Familial Apo C-II Deficiency

The clinical manifestations of familial apo C-II deficiency are identical to those of familial LPL deficiency. However, as a small part of LPL is activated independently of apo C-II, the symptoms are usually moderate and the diagnosis is often belated. The disease generally comes to light in adult patients and rarely during childhood, with acute pancreatitis [33].

### 4.4. Primary Hyperlipemia Type V

Primary hyperlipemia type V is expressed with chylomicronemia and mild increase of VLDL plasma levels [34]. Families suffering from primary hyperlipemia type V frequently present genetic alterations as familial combined hyperlipidemia and familial hypertriglyceridemia. Furthermore, environmental diseases, like diabetes, alcoholism, and obesity, are considered as a cause of primary hyperlipemia type V. Acute pancreatitis may occur in 17% of patients [35].

## 5. SEHTG Treatment

SEHTG treatment is based on strict dietary measures and on correcting secondary factors and unhealthy lifestyle habits, such as lack of exercise. Drug therapy is indicated for patients with mild HTG as recommended by NCEP ATPIII guidelines [12]. The treatment of HTG other than LDL apheresis will be only briefly mentioned because it is beyond the scope of this review.

### 5.1. Dietary Therapy

Dietary therapy is based on restriction of fat consumption, recommending daily fat intake of less than 20 gr (corresponding at 15% of total daily calories), leading thus to TG concentrations ~1500 mg/dL [36]. Infants and children should be given fat free milk with medium chain triglycerides (MTCs), entering the circulation without prior necessity of chylomicrons incorporation [37]. During the 2nd and 3rd trimester, fat intake may be limited to 2 gr daily, with no risk to the embryo [38]. Acute pancreatitis is treated with fasting and insulin and heparin administration [39]. Further, weight loss, as a result of a dietary regimen, provokes reduction in TG concentrations [40–42].

Actually, a single 10% weight loss is capable of reducing postprandial TG concentrations, even in obese patients, increasing remarkably the insulin sensitivity [43].

### 5.2. Physical Activity

Physical activity presents positive, though brief effect on TG decrease, suggesting the need for systematic daily training of 30–60 minutes or at least 150 minutes weekly [44].

Intermittent training also seems as an adequate alternative for sedentary patients, while isometric exercise increases the lean body mass and reduces the adipose tissue.

### 5.3. Drug Therapy

Statins are the cornerstone of treatment, followed by fibrates and n-3 fatty acids, to achieve recommended therapeutic levels of plasma LDL cholesterol, non-HDL cholesterol, and apo B-100 if HTG is mild. The case for using niacin has been weakened by the results of clinical trials but needs further investigation. According to SEHTG the* LPL gene* replacement therapy is an option (in familial LPL deficiency) and new inhibitors, such as diacylglycerol O-acyltransferase, MTP, and apo C-III antisense oligonucleotides, are presenting valuable weapons; however, they are still under investigations [45, 46]. In cases with symptomatic SEHTG the LDL apheresis becomes a very important tool.

LDL apheresis is often required starting from a young age. Apheresis can lower LDL cholesterol levels by 80% acutely and 30% chronically (weekly or biweekly) [47, 48] and TGs by 30–50%.

## 6. LDL Apheresis Treatment in Patients with SEHTG Original Findings

Five patients with SEHTG underwent the LDL apheresis in Onassis Cardiac Surgery Center.

### 6.1. Methods

In the present study we selected patients with SEHTG with risk of acute pancreatitis who experienced at least one episode of pancreatitis. Patients underwent LDL apheresis procedure (see below) any time they had TG concentrations >1000 mg/dL (Table 1). This was checked routinely 3-4 times annually in the outpatient bases by blood sample. In case of abdominal pain the additional measurement was required. There were not any exclusion criteria.

The diabetes mellitus was diagnosed if the fasting glucose was ≥126 mg/dL or patient was under antidiabetic treatment.

The hypertension was diagnosed if systolic blood pressure was >140 mmHg and/or diastolic blood pressure was >90 mmHg or patient was under antihypertensive treatment.

The body mass index was calculated as weight divided by height squared (BMI: body weight/height squared).

The LDL apheresis procedure was described in detail elsewhere [49]; briefly, the DALI 500, 750, 1000 (500 + 500), and 1250 (500 + 750) adsorbers, blood lines, and hemoadsorption monitor 4008 ADS (Fresenius HemoCare Adsorber Technology GmbH, St. Wendel, Germany) were used. Two bilateral vascular accesses by venipuncture, generally in the median cubital veins, were established. At the start of the session the patient was only connected to the afferent (arterial) line of the extracorporeal circuit. All sessions were carried out under blood pressure and electrocardiogram monitoring. DALI 750–1250 adsorbers were used. The main indication for use of the DALI system was symptomatic (acute pancreatitis) in spite of proper diet and triglyceride lowering drugs. The laboratory measurements for the lipid profile were drawn at the start (before any procedure) and end (just before taking of the needle from the afferent arm) of each session. Blood was collected in tubes containing EDTA. Plasma TC, TG, and high density lipoprotein (HDL) cholesterol levels were measured using enzymatic colorimetric methods on a Roche Integra Biochemical analyzer with commercially available kits (Roche). All samples were analyzed within few hours.

The LDL apheresis procedure is approved by ethic committee of Onassis Cardiac Surgery Center and since it is an invasive procedure all patients sign the informed consent.

### 6.2. Statistical Analysis

The results were expressed as mean ± standard deviation (SD). Student's *t*-test was used to compare the continuous variables between groups, at a significance level of *P* < 0.05 (Table 2, Table 3).

The acute differences in TG concentrations before and after LDL apheresis were described as % difference, based on the following rule: % difference = [(variable before − variable after)/variable before] ∗ 100. The LDL cholesterol was not calculated due to very high baseline TG concentrations (>4.5 mmol/L (400 mg/dL).

### 6.3. Results

Clinical characteristics of the patients are presented in Table 1. Since the TG concentrations were >400 mg/dL and we have not performed direct LDL cholesterol measurement the levels of LDL cholesterol were not provided.

No renal dysfunction was presented. We perform LDL apheresis for nearly 11 years. The length of follow up period differed among patients because cases of SEHTG and risk of pancreatitis are uncommon. All treated patients remain under surveillance.

The medication list shows the current medication. Patients who were under angiotensin converting enzyme inhibitors prior to entering the DALI sessions were switched to calcium-channel blockers for controlling the blood pressure. No other major changes according to drug treatment were made.

Two patients experienced cardiovascular events: 1 underwent cardiac death (50-year-old men) independently of the sessions and 1 underwent percutaneous coronary intervention angioplasty. Three patients presented new episodes of acute pancreatitis. Annual average event rate of 6% was found.

## 7. LDL Apheresis and SEHTG: Review of the Literature

Apheresis for lowering TG concentrations was first performed by Betteridge et al. [50] in 1978. Since then the plasmapheresis has been a therapeutic tool for SEHTG also. Currently available studies have proved that apheretic treatment is effective by lowering significantly and rapidly TG concentrations [51].

A multicenter study of 17 patients with SEHTG nonresponders to conventional medical therapy reported by Stefanutti et al. [52] showed that the removal of TG-rich lipoproteins by plasmapheresis prevented relapses of acute pancreatitis and this kind of intervention has been confirmed as a safe and reliable method.

Another very important issue according the apheresis treatment is the timing of the apheretic treatment. Several reports showed that maximal reduction in morbidity and mortality can be achieved when apheresis is used as early as possible [51, 53].

In 1993, Bosch et al. first described LDL direct adsorption of lipoproteins (DALI), Fresenius, Germany [54–61]. The LDL apheresis treatment is offered by a limited number (40–50) of centers in the United States. In Greece this kind of intervention was limited to 1 center, and only recently 2 more were added.

Furthermore, there are inadequate data concerning LDL apheresis treatment and SEHTG. Single nonrandomized controlled trial and case reports have examined the use of plasmapheresis to treat acute pancreatitis due to HTG [62–65]. Approximately the 40–80% of TGs reduction has been reported with symptoms relief of pancreatitis following one to three plasmapheresis procedures. The single trial (with historic control) by Yeh et al., however, found no difference between standard therapy and plasmapheresis versus standard therapy alone in patients with severe acute pancreatitis with regard to mortality, systemic complications, and local complications in patients with severe pancreatitis [66]. Adequate information was not provided to ascertain the comparability of the two groups. While the authors felt that these negative findings were due to delayed initiation of plasmapheresis and recommended earlier intervention, the time from diagnosis to start of plasmapheresis was not provided.

Eight case reports examined plasmapheresis use in pregnant women with HTG-induced pancreatitis. In six cases, plasmapheresis was performed due to the presence of pancreatitis [67–72]. The number of treatments ranged from 1 to 10 (median 2) with Cesarean section due to fetal distress and delivery of a preterm infant occurring in 5 of 6 cases. Since the treatment with fibrates, the mainstay of medical therapy for HTG, has been associated with teratogenic effects, plasmapheresis had been used as an alternative prevention and treatment strategy during pregnancy. In two additional cases, patients were treated prophylactically because of a history of pancreatitis. Plasmapheresis was performed 6 and 13 times beginning at 25 and 19 weeks' gestation, respectively. In both cases, healthy infants were delivered at 34 weeks. In one of these cases, treatment was determined by TG concentrations with a goal to maintain a TG below 1,000 mg/dL.

Two case reports have examined plasmapheresis in generalized lipoatrophy. Serial plasmapheresis was used to control HTG and avoid pancreatitis [73]. One report found benefit while one did not. In the latter, a variety of metabolic abnormalities were noted following plasmapheresis, including amenorrhea, galactorrhea, proliferative retinopathy, and hypertension which were attributed to the treatment. The authors did not recommend plasmapheresis because of these findings. It should be noted that these complications have not been reported as complications of plasmapheresis and are therefore of questionable association.

Other causes of HTG pancreatitis that have been reported to be treated by plasmapheresis include HTG due to medications such as isotretinoin, ritonavir, cyclosporine, and asparaginase as well as case report of lipid emulsion overdose in a patient on total parenteral nutrition [74–76]. In all of these cases, treatment has been reported to be beneficial.

Two series have reported chronic plasmapheresis treatment in a total of 8 patients with recurring pancreatitis [62, 63, 77]. Both series reported TPE reduced or prevented further episodes of pancreatitis. In one of these two series, (6 patients), the frequency of pancreatitis was reduced by 67%. Treatments were done at a frequency to maintain the TG concentrations below 150 mg/dL.

It is important to note that plasmapheresis can be effective to rapidly decrease the TG level; its effect is transient; adequate lipid lowering treatment is essential to achieve the persistent effect [72].

## 8. Discussion

As was already analyzed above, the general treatment regimen for SEHTG includes dietary restrictions and lipid lowering drug treatment. However, fibrates (first line drug for treatment of SEHTG) do not offer fast onset of action, while immediately acting drugs like omega-3-fatty acids and MCT may not lower sufficiently the excessive increased TG concentrations. Since patients with SEHTG are in urgent need of fast and effective lowering of their TG concentrations in order to prevent a severe pancreatitis episode, further therapeutic steps such as apheresis treatment should be performed. Although plasmapheresis as a treatment for HTG was first described nearly 35 years ago [50], it remains poorly evaluated in the literature, most likely due to its accessibility and expensiveness. Because the diseases treated with apheresis are uncommon, the reported evidences are approaching case reports or small case series. Thus, no randomized controlled trials have entered PubMed in English on this specific topic. The American Society of Apheresis (ASFA) guidelines refer to plasmapheresis for HTG in the context of pancreatitis [80].

In the SEHTG state the urgent aim is to reduce endogenous and exogenous sources of TGs and stimulate their clearance. Exogenous sources can be reduced by low-fat diets and fasting; endogenous sources can be reduced by lipid lowering medications and limited alcohol intake. Plasmapheresis has emerged as an effective tool in rapidly reducing plasma TG concentrations and helping to treat and prevent HTG-induced pancreatitis.

Most reports on apheresis associated with HTG deal with plasmapheresis, which typically lowers TG concentrations by 60–70% in one session [66]. However, single and double filtration plasmapheresis do not selectively remove lipoproteins and TG but remove also coagulation factors and immunoglobulins with possible adverse events such as infections. Thus, we chose an approach with selective lipoprotein apheresis, although the treatment is not as effective for lowering TGs as the plasmapheresis techniques. Furthermore, all patients had elevated plasma total cholesterol in spite of the hypolipidemic drug treatment, which also makes them candidate to LDL apheresis treatment.

We provided data of 5 patients who underwent LDL apheresis due to SEHTG and risk of acute pancreatitis. LDL apheresis proved to be effective and after the procedure the TG concentrations decreased acutely additionally to diet and drug treatment by 25–35%. However, the major cardiovascular events and recurrence of pancreatitis, which were reported, were very high. This may be explained that patients were all men presenting with cardiovascular disease (2 patients), with history of pancreatitis (all patients), and with risk factors for cardiovascular disease such as obesity (all patients), hypertension (2 patients), and diabetes mellitus (2 patients). Additionally, the SEHTG is a chronic disease and many times patients are tired of very strict diet and alcohol restriction.

The limitation of the finding is the small size sample.

## 9. Conclusions

The LDL apheresis for very severe or extreme high TG concentration should be recommended only under very uncommon (usually symptomatic) clinical conditions unless more evidence will be provided.

## Figures and Tables

**Table 1:** Clinical characteristics of 5 patients undergoing LDL apheresis sessions.

Demographic characteristics					
Age, years	44	46	55	59	46
Men	Y	Y	Y	Y	Y
BMI	33.6	32.1	38.2	33.3	34.0
Hypertension	Y	N	N	Y	N
Diabetes mellitus type 2	Y	N	N	Y	N
Smoking	N	N	N	N	N
Previous conditions					
Pancreatitis	1	4	1	1	1
Myocardial infarction	N	N	N	Y	N
Coronary artery bypass grafting	Y × 2	N	N	Y	N
PCI angioplasty	Y	N	N	N	N
Cardiac failure	Y	N	N	Y	N
Beta-blockers	Y	N	N	Y	N
Nitrates	Y	N	N	N	N
Calcium-channel blockers	N	N	N	Y	N
Statins	Y	Y	Y	Y	N
Fibrates	Y	Y	Y	Y	Y
Ezetimibe	Y	N	N	N	N
Omega-3	Y	N	Y	Y	Y
Aspirin	Y	N	N	Y	N
Clopidogrel	Y	N	N	Y	N
Levosimendan	Y	N	N	Y	N
Diuretics	Y	N	N	Y	N
Followup (months)	132	12	36	80	28

PCI indicates percutaneous coronary intervention, ACE indicates angiotensin converting enzyme, Y indicates yes, and N indicates no.

**Table 2:** Lipid profile before and after LDL apheresis sessions in all cases.

Variable	Before LDL apheresis	After LDL apheresis	% difference
	Mean ± SD/median (range)	Mean ± SD/median (range)	Mean ± SD/median (range)
TC	330.3 ± 176.0	180.0 (770.0–71.0)	31.3 ± 15.6
TG	1754.0 (7792.0–1015.0)	1121.0 (5976–100)	32.33 (100.0–1.6)
HDL-C	34.7 ± 17.2	29.6 ± 16.6	15.0 (66.7–127.3)

All data are expressed as mg/dL, mean ± SD/median (range). The mean data are based on multiple measurements (*n* = 102, only sessions, which had all three parameters, TC, TG, and HDL-C, measured, were involved) during all the followup period. All values were significantly decreased *P* < 0.001. To convert cholesterol values (TC, HDL-C) from mg/dL to mmol/L, multiply by 0.0259 and, for TGs, multiply by 0.0113. TC indicates total cholesterol; TG indicates triglycerides; HDL-C indicates high density lipoprotein cholesterol.

**Table 3:** Lipid profile before and after LDL apheresis sessions by each patient.

Pt. ID	Lipids	Before LDL apheresis	After LDL apheresis	% difference
		Mean ± SD/median (range)	Mean ± SD/median (range)	Mean ± SD/median (range)
1	TC	366.6 ± 144.0	196.0 (308.0–90.0)	40.8 ± 12.4
	TG	1427.0 (5781.0–1024.0)	944.5 (4462.0–497.0)	35.21 (54.6–21.6)
	HDL-C	51.3 ± 10.5	46.9 ± 16.2	12.5 (30.2–(−23.6))

2	TC	395.5 ± 95.5	288.5 (364.0–213.0)	28.2 ± 9.7
	TG	3137.5 (3888.0–2387.0)	2070.0 (2753.0–1387.0)	35.54 (41.9–29.2)
	HDL-C	37.0 ± 2.8	27.5 ± 4.9	24.95 (38.5–11.4)

3	TC	492.0 ± 167.8	355.0 (770.0–162.0)	20.7 ± 17.7
	TG	3238.0 (7792.0–1200.0)	2375.0 (5976.0–488.0)	24.36 (85.3–1.6)
	HDL-C	40.6 ± 17.1	33.1 ± 15.4	28.0 (66.67–(−127.27))

4	TC	212.04 ± 27.53	120.0 (167.0–101.0)	40.2 ± 8.9
	TG	1279.0 (2031.0–1015.0)	773.0 (1469–100)	39.3 (100.0–19.0)
	HDL-C	29.9 ± 4.9	25.3 ± 2.8	14.3 (30.0–6.7)

5	TC	168.4 ± 31.5	121.0 (205.0–75.0)	26.72 ± 8.33
	TG	1741.0 (2537.0–1314.0)	1339.0 (2057–100)	25.7 (100.0–7.2)
	HDL-C	11.6 ± 1.9	10.0 ± 1.7	11.11 (27.3–(−7.7))

All data are expressed as mg/dL, mean ± SD/median (range). The mean data are based on multiple measurements (*n* = 102, only sessions, which had all three parameters, TC, TG, HDL-C, measured, were involved) during all the followup period. All values were significantly decreased *P* < 0.001. To convert cholesterol values (TC, HDL-C) from mg/dL to mmol/L, multiply by 0.0259 and, for TGs, multiply by 0.0113. Pt. ID indicates patient identification; TG indicates triglycerides; HDL-C indicates high density lipoprotein cholesterol.
